# Examining the Gastrointestinal Effects of Solnul^®^, a Prebiotic Resistant Potato Starch: A Replication Randomized, Double-Blind, Placebo-Controlled Clinical Trial in Healthy Adults

**DOI:** 10.3390/nu18213538

**Published:** 2026-10-27

**Authors:** Jason R. Bush, Izuchukwu Iwuamadi, Edward C. Deehan

**Affiliations:** 1 MSP Starch Products Inc., Carberry, MB R0K 0H0, Canada; 2 Department of Food Science and Technology, University of Nebraska, Lincoln, NE 68588, USA; iiwuamadi2@huskers.unl.edu; 3 Nebraska Food for Health Center, University of Nebraska, Lincoln, NE 68588, USA

**Keywords:** *Akkermansia*, *Bifidobacterium*, Bristol Stool Form Scale, clinical trial, dietary fiber, prebiotic, resistant potato starch

## Abstract

Background: A prebiotic is a substrate that is selectively utilized by host microbiota to confer a health benefit. Consumption of 3.5 g of Solnul^®^ resistant potato starch (RPS) was previously shown to selectively increase *Bifidobacterium* and *Akkermansia*, as well as improve stool form relative to a placebo. The objective of this study was to reproduce these effects in an independent cohort. Methods: A 4-week, randomized, double-blind, placebo-controlled, parallel group trial evaluated the effects of 3.5 g RPS versus a placebo on changes in *Bifidobacterium*, *Akkermansia*, stool form, and bowel movement (BM) frequency using 16s rRNA sequencing, the Bristol Stool Form Scale, and personal diaries in healthy adults aged 18–69 years in Guelph, ON. Results: Compared to the placebo (n = 35), the relative abundance of *Bifidobacterium* increased (*p* = 0.049, Brunner–Munzel), and the number of normal stools (Type 3 or 4) was higher in the RPS group (n = 35; *p* = 0.0077, Chi-Square; key secondary endpoints). While differences were not observed for *Akkermansia* relative to the placebo (*p* = 0.49; primary endpoint), End-of-Study *Akkermansia* levels were higher than Run-In levels for RPS (*p* = 0.046, Wilcoxon signed-rank) but not the placebo (*p* = 0.14). Participants consuming RPS had higher BM frequency at the End-of-Study compared to the Run-In period, but the effect was not different from the placebo. Comparing End-of-Study and Run-In periods, the RPS group experienced fewer days without a BM relative to the placebo. No changes in the GSRS or serious adverse events were reported in the RPS group. Conclusions: This clinical trial confirmed that 3.5 g of RPS induces a bifidogenic effect and normalized stool form, consistent with the previous study. NCT06949631.

## 1. Introduction

As defined by the International Scientific Association for Probiotics and Prebiotics (ISAPP), prebiotics are substrates selectively utilized by host microorganisms that confer a health benefit [1]. To satisfy the requirements of this definition, clinical trials evaluating candidate prebiotics must demonstrate evidence of selective utilization by host microorganisms, which typically involves demonstrating an increase in the abundance of bacteria known to use the substance as a substrate, as well as an improvement in the health status of the participants. Elaborating on the criteria for prebiotic substantiation, ISAPP identified confirmatory evidence in the form of additional trials as an optional final step to increase confidence in prebiotic efficacy [2].

Despite this recommendation, research programs aiming to reproduce prebiotic outcomes for an ingredient typically conduct clinical trials in different populations, at different ingredient dosages, over varied time courses. For example, chicory-derived inulin-type fructans increase *Bifidobacterium* abundance and promote beneficial effects on measurements of bowel function at doses of 3–20 g per day [3]. True reproducibility is more difficult to establish: A pair of clinical trials examining 12 g of chicory inulin per day for 4 weeks failed to reproduce improvements in stool form or increases in *Bifidobacterium* [4,5,6]. Trials that reproduce the effects of an ingredient while evaluating the same dose for the same duration in the same population have emerged as a gold standard for clinical research in the prebiotics industry.

Resistant starch (RS) refers to “the sum of starch and starch degradation products that, on average, reach the human large intestine” [7]. The types of RS are categorized on the basis of their resistance: Type 1 are physically inaccessible, Type 2 resist digestion via the natural crystallinity of their granules, Type 3 are retrograded, Type 4 are chemically modified, and Type 5 are complexed with lipids [8]. Different sources of RS produce different effects on the gut microbiota, even within the same RS type. For example, corn RS4 stimulated increases in *Ruminococcus* spp., tapioca RS4 stimulated *Parabacteroides* spp., and potato RS4 did not influence the composition of the gut microbiota [9]. For these reasons, clinical studies demonstrating the benefits of a specific form of RS cannot be generalized to RS ingredients from other sources.

Resistant potato starch (RPS) is one of the most clinically studied prebiotic RS ingredients. *Bifidobacterium* related bacteria are primary degraders of RPS [10,11,12], and dietary supplementation with RPS generally increases the relative abundance of *Bifidobacteria* in people [13,14,15,16]. In addition to increasing stool butyrate levels [13,14,17], prebiotic-associated health benefits of RPS include laxation [18], stool form normalization [15], metabolic improvements [14,19], and nutrient absorption [20,21]. While these studies establish RPS as a prebiotic, clinical trials evaluating the same dose, for the same duration, in the same population have not been reported.

The primary objective of the present study was to test whether RPS stimulated a greater increase in *Akkermansia* compared to a placebo. Secondary objectives included reproducing the bifidogenic and stool normalization effects that were previously reported in a clinical trial evaluating daily consumption of 3.5 g of RPS for 4 weeks [15]. Changes in bowel movement frequency and days without a bowel movement were also compared as exploratory measures. To facilitate direct comparisons, data from the previous trial was reanalyzed using a similar statistical framework as exploratory measures.

## 2. Materials and Methods

### 2.1. Investigational Product

The clinical trial aimed to determine the reproducibility of the beneficial gastrointestinal effects previously demonstrated by Bush and colleagues [15] for prebiotic resistant potato starch (RPS) supplementation. Specifically, the present study investigated the gastrointestinal effects of consuming 7 g/day of total starch, which comprised 3.5 g of RPS (Solnul^®^, MSP Starch Products Inc., Carberry, MB, Canada) and 3.5 g of high-amylopectin corn starch (Amioca TF; Ingredion, Brampton, ON, Canada). Solnul^®^ is a Type 2 RS that is produced via a unique proprietary processing method that preserves starch granule structure, resulting in stable RS contents of ≥60% (AOAC 2002.02). Every 3.5 g dose of Solnul^®^ contains ≥2.1 g of RS on an ‘as-is’ basis. The Solnul^®^ lot used in this study contained 61.4% RS on an ‘as-is’ basis (Lot 008546; AOAC 2002.02). Therefore, each participant in this study consumed 2.1 g of RS per day. In contrast to the treatment, the placebo contained 7 g of high-amylopectin corn starch (Amioca TF). The placebo was previously shown to be fully digestible starch with no RS [22], with no effects on the gut microbiota even when supplemented at high amounts of 50 g/day [9]. The RPS and placebo doses were matched for caloric content (23.9 kcal and 25.1 kcal, respectively), were nearly identical in appearance and taste, and were packaged in identical single-serving sachets using randomization codes generated from Nutrasource SGS (Guelph, ON, Canada; see Section 2.2 for details) by personnel not involved in the collection of study data to ensure blinding of the study.

### 2.2. Study Design and Participant Randomization

**Figure 1:**
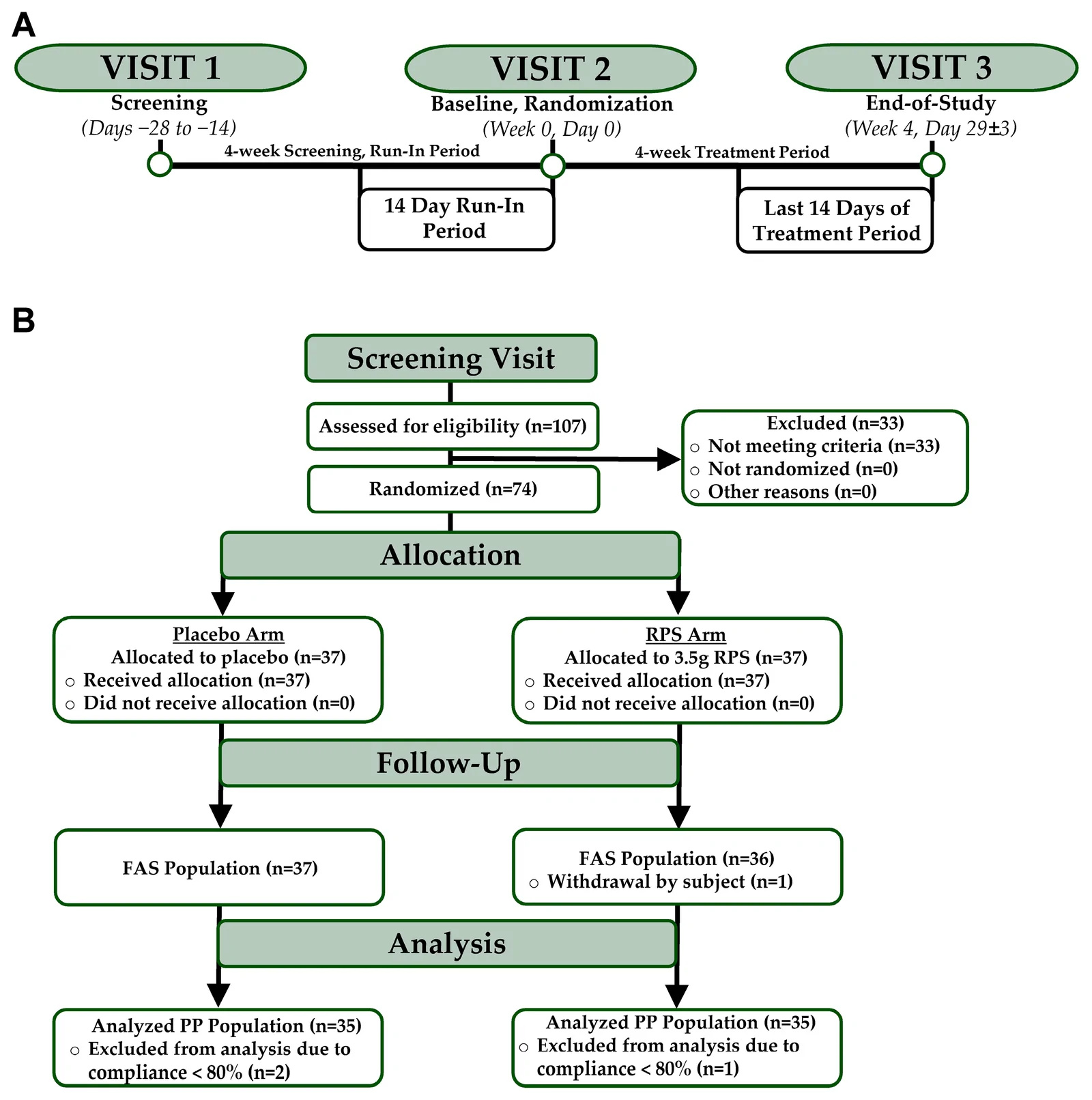
Clinical trial design with a CONSORT flow diagram. (**A**) Randomized, double-blind, placebo-controlled clinical trial design. (**B**) The CONSORT flow diagram shows that 107 individuals were assessed for eligibility, of which 74 were randomized to the RPS or placebo arm. Full analysis set (FAS) and per-protocol (PP) populations are indicated, including reasons for participant attrition.

The randomized, double-blind, placebo-controlled, parallel-armed clinical trial was conducted at Apex Trials (Guelph, ON, Canada) between 14 May 2025 and 3 October 2025 in Guelph, ON, Canada (Figure 1A). The study protocol, informed consent form, and related documents were approved by the Sterling IRB (IRB Registration Number: 00001790; Protocol M03-25-01-T0081; Atlanta, GA, USA) on 25 April 2025, with the trial registered on 22 April 2025 at ClinicalTrials.gov (NCT06949631). The clinical trial was conducted in accordance with the IRB-approved protocol and with the consensus ethical principles derived from international guidelines, including the Declaration of Helsinki and Council for International Organizations of Medical Sciences International Ethical Guidelines, applicable International Council for Harmonization (ICH) Good Clinical Practice Guidelines (accessed on 1 April 2025) [23], and applicable local and federal laws and regulations. Onsite monitoring was contracted to Nutrasource SGS (Guelph, ON, Canada), whose clinical trial management team ensured the clinical trial was conducted according to the approved clinical monitoring plan. Data management was provided by Nutrasource SGS using the electronic data capture system Medrio (San Francisco, CA, USA; Version R42.0). Study conduct, oversight and analyses were conducted in accordance with Health Canada Natural Health Product Regulations and guidance documents (accessed on 1 April 2025) [24].

The sample size calculation used a two-sided *t*-test with α = 0.05 and β = 0.8, with an expected difference in mean of 0.03639 and pooled standard deviation of 0.052613 based on *post hoc* analysis of *Akkermansia* changes from the previous clinical trial [15]. The sample size goal was set at 74 participants to allow for an anticipated attrition rate of 8%. Prior to participant enrollment, a block randomization scheme was generated by Nutrasource SGS using SAS 9.4 PROC PLAN on 10 April 2025 (Seed Number: 2059911900). The randomization scheme consisted of 40 blocks, with each block containing two participants, of which only 37 blocks were used. The clinical trial included a screening visit from 28 days to 14 days prior to randomization (Visit 1), a 14-day Run-In period followed by a baseline visit at Week 0 (Visit 2), during which the randomization was performed, and an End-of-Study visit at Week 4 (Visit 3; Day 29 ± 3).

During the screening visit (Visit 1), a bowel habits diary, a food record diary, and stool collection instructions and materials were provided, which included fecal sample collection containers and OMNIgene-Gut kits (DNA Genotek, Ottawa, ON, Canada). During the 14-day Run-In period, participants recorded their daily bowel habits and food intake in the diaries, as well as collected a fecal sample within 72 h prior to the baseline visit per DNA Genotek’s instructions and transferred it to the clinic within 3 days of collection. During the baseline visit (Visit 2), participants completed the Gastrointestinal Symptom Rating Scale (GSRS) questionnaire [25]. Then, participants were instructed to mix one sachet of the randomly assigned starch treatment with approximately 125 mL of cool or room temperature water, drink the mixture immediately before the test product settled, and then add a bit of extra water to ensure all the product was consumed and no starch residue remained. Participants were instructed to consume the investigational product once daily in the morning for the duration of the study. At Visit 2, participants were also provided with bowel habit and food record diaries, GSRS questionnaires, stool collection supplies, and a 35-day supply of the investigational product for the 4-week Treatment period. Finally, during the last 14 days of the Treatment period, participants recorded their daily bowel habits and food records in the diaries, as well as collected a fecal sample within 72 h prior to the End-of-Study visit (Visit 3). Participants returned all sachets, including any open or unopened sachets, at Visit 3. Protocol adherence for this study was considered acceptable if participants consumed >80% of the test product expected for the given study duration and at least 25 sachets of test product were consumed. Furthermore, adherence to habitual dietary habits was monitored using food records by averaging three days recorded near the end of the Run-In and Treatment periods.

### 2.3. Participant Selection and Enrollment

Study participants were recruited using the Apex Trials’ participant database, as well as print and electronic advertisements for the general population in Guelph and the surrounding area. Participants enrolled in this study were adults (18 to 69 years of age, inclusive) with a BMI of 18.0 to 34.9 kg/m^2^, who were considered to be in good general health (i.e., no uncontrolled diseases or conditions) as determined by the investigator based on medical history and screening bloodwork. Participants had no gastrointestinal disorders, including irritable bowel syndrome (IBS) or dyspepsia, and no conditions such as lactose and/or gluten intolerance, proneness to rectal bleeding due to hemorrhoids, or persistent gastrointestinal problems. Participants agreed to maintain consistent dietary habits, including medication and supplement intake, and lifestyle activities for the last 3 months before screening and throughout the study, with the exception that participants were to refrain from alcohol and moderate to high-intensity activities for 24 h prior to each study visit. Furthermore, participants agreed to follow the restrictions on concomitant treatments and the restrictions on lifestyle and to use acceptable contraceptive methods.

The study investigator or their representative designee explained the nature of the study to each participant and answered all questions regarding the study. Participants were informed that their participation was voluntary. If they wished to participate in the study, they were required to sign a statement of informed consent that met the requirements of local regulations, ICH guidelines, and the IRB. The source document included a statement that written informed consent was obtained before each participant was enrolled in the study and the date the written consent was obtained. The authorized person obtaining the informed consent also signed the ICF. Only after providing this consent were initial medical evaluations conducted. These activities were completed prior to enrollment. Therefore, all enrolled participants were willing and able to agree to the requirements of this study, provide voluntary consent, understand and read the questionnaires, and carry out all study-related procedures.

### 2.4. Safety

The FAS (n = 73) consisted of all participants who received at least 1 dose of the study product. Treatment-emergent adverse events (TEAEs) were defined as adverse events (AEs) with the onset time on or after the first dose of the study product. The relationship between the study intervention and each AE was assessed by the study clinician using their best judgment by examining and evaluating the participant based on the temporal relationship to the AE.

### 2.5. Gastrointestinal Symptom Rating Scale (GSRS)

Participants completed the validated GSRS questionnaire based on the last 7 days before the baseline visit (Visit 2) and the End-of-Study visit (Visit 3) [26]. The gastrointestinal symptoms were scored on a 7-point Likert scale, where 1 = No discomfort at all, 2 = Minor discomfort, 3 = Mild discomfort, 4 = Moderate discomfort, 5 = Moderately severe discomfort, 6 = Severe discomfort, and 7 = Very severe discomfort. Subscale scores were calculated for reflux, abdominal pain, indigestion, diarrhea, and constipation, as well as a Total GSRS Symptom Score.

### 2.6. Bristol Stool Form Scale and Bowel Movement Frequency

Participants scored their bowel movements using the Bristol Stool Form Scale (BSFS; also known as the Bristol Stool Chart), where Type 1 = constipation with hard, round stools, Type 2 = lumpy and sausage-like stools, Types 3 and 4 are soft, easily passed ‘normal’ stools, Type 5 = soft blobs of stool with clear-cut edges, Type 6 = stools with mushy consistency and ragged edges, and Type 7 = watery diarrhea [27]. This scoring was done during the last seven recorded bowel movements to align with the microbiome analysis at Week 4, which was based on a stool sample collected 72 h prior to Visit 3, consistent with the previous clinical trial [15].

Daily bowel movements were also recorded for each participant during the 14-day Run-In period and during their last 14 days of the Treatment period. The records were used to assess changes in bowel movement frequency and calculate changes in the number of days without a bowel movement, with missing records assumed to be a day without a bowel movement. Imputed data was then generated for participants with missing records: For each participant with missing records, the number of days with a BM during the recorded days was divided by the number of recorded days to determine the likelihood of that participant having a BM on a missing day, the value rounded to 1 or 0 and then multiplied by the number of missing record days. Similarly, for each participant with missing records, the number of BMs recorded was divided by the number of recorded days to estimate the number of BMs that participant likely had on a missing record day; then, the values were multiplied by the number of missing record days.

### 2.7. Microbiota Analysis

Fecal samples were collected in OMNIgene-Gut kits (DNA Genotek, Stittsville, ON, Canada), and DNA was extracted using the NucleoMag DNA Microbiome kit (Machery-Nagel, Allentown, PA, USA) using a KingFisher robot (ThermoFisher, Waltham, MA, USA). The quality of the extracted DNA was evaluated visually via gel electrophoresis and quantified using a Qubit 3.0 fluorometer (ThermoFisher). Using PCR primers that targeted the V4 region of the 16S rRNA gene (515F 5′-GTGCCAGCMGCCGCGGTAA-3′ and 806R 5′-GGACTACHVGGGTWTCTAAT-3′) [28], 16S rRNA gene amplicons were generated and sequenced on a MiSeq platform (Illumina, San Diego, CA, USA) at Microbiome Insights (Richmond, BC, Canada). The microbiota analysis aimed to replicate the methods previously described by Bush and colleagues [15]. Briefly, raw Fastq files were quality-filtered, aligned to the Silva master alignment v132 and clustered into 97% similarity operational taxonomic units (OTUs) using the mothur software package v1.48.0 [29]. High-quality reads were classified using the Silva reference database (Silva.nr v138_1). A consensus taxonomy for each OTU was obtained, OTU abundances were aggregated into 7451 taxonomies, and the relative abundances were plotted. An average of 34,463 quality-filtered reads was generated per sample. Sequencing quality for reads one (R1) and two (R2) was determined using FastQC. *Bifidobacterium* and *Akkermansia* were identified *a priori* as candidate genera expected to increase in response to RPS consumption [15]. In addition to genera, phyla (*Actinomycetota* and *Verrucomicrobiota*), families (*Bifidobacteriaceae* and *Akkermansiaceae*), and the most abundant representative OTUs (average relative abundance >0.01%) were included in the statistical analysis.

### 2.8. Statistics

All statistical analyses were performed using Microsoft Excel (Version 2608, Redmond, WA, USA), and *p* < 0.05 was considered statistically significant. To control for outliers in the 16Sv4 data set, the relative abundances of bacterial taxa were log-transformed and absolute z-scores generated [30]. Samples exceeding ±2 standard deviations were excluded prior to analysis. Changes in bacterial taxa relative abundance in response to the RPS and placebo were first tested for normality using the Shapiro–Wilk test and were found to exhibit a non-normal distribution. Changes in bacterial taxa relative abundance were then tested for heteroscedasticity using Levene’s test. Bacterial taxa data that tended to show heteroscedasticity were compared between treatments using the Brunner–Munzel test [31]. Bacterial taxa data that did not show heteroscedasticity were alternatively compared between treatments using the Mann–Whitney U test. Within-treatment-group comparisons were made using the Wilcoxon signed-rank test using Pratt’s signed-rank zero procedure to accommodate ties, followed by Bonferroni corrections [32]. Associations between stool form type and treatment type during the last seven bowel movements before the End-of-Study visit of the trial were assessed using Chi-Square analysis. Given that repeated stool observations do not account for within-participant dependence, the number of participants who experienced normal stools was compared to those who did not using Chi-Square analysis. Differences between GSRS scores, macronutrient intake, bowel movements per day, and number of days without a bowel movement were compared using Student’s *t*-test with Bonferroni corrections (within-group comparisons only).

## 3. Results

### 3.1. Participant Characteristics

A total of 107 participants were screened for eligibility, of whom 74 were enrolled and randomly assigned to consume either the RPS or placebo treatment daily for 4 weeks (Figure 1B). One participant in the RPS arm withdrew at the end of the Run-In period and did not receive any test product (Figure 1B). The baseline demographic information of the enrolled study participants is shown in Table 1. Three participants were excluded from analysis due to poor adherence to the study protocol, including one participant in the RPS group (20 sachets used; 74.1% compliance) and two in the placebo group (24 sachets used; 88.9% compliance; 24 sachets used; 77.4% compliance; Figure 1B). Thus, analysis of safety profiles was performed using the full analysis set (n = 74), while all remaining analyses were performed using the per-protocol population (n = 70) that included only those participants who completed the full study protocol in adherence with all study parameters. Despite the enrollment of generally healthy participants and the exclusion of individuals with gastrointestinal-related medical diagnoses, participants in both treatment arms experienced bowel movements corresponding to all seven stool types according to the BSFS during the Run-In period (Supplementary Table S1).

### 3.2. Safety

There were similar incidences of treatment-emergent adverse events (TEAEs) in the RPS and placebo groups. Thirteen participants experienced 18 TEAEs during the study period (Table 2). The TEAEs were diverse in nature, with five participants reporting instances of gastrointestinal symptoms, four participants reporting instances of musculoskeletal pain, three participants reporting instances of common cold symptoms, one participant reporting light sensitivity and dizziness, one participant reporting stress, and one participant reporting presyncope. Two TEAEs were assessed by the principal investigator (PI) as being possibly related to the study products, and both were reports of abdominal bloating in participants consuming the placebo. All TEAEs were resolved, and there were no serious adverse events or deaths during the study period.

### 3.3. Macronutrient Intake

Macronutrient intake changes were monitored using food records by averaging three days recorded near the end of the Run-In and Treatment periods. Neither the RPS group nor the placebo group showed changes in reported macronutrient intake during the study (*p* > 0.05; Supplementary Table S2).

### 3.4. Gastrointestinal Symptom Rating Scale (GSRS)

**Figure 2:**
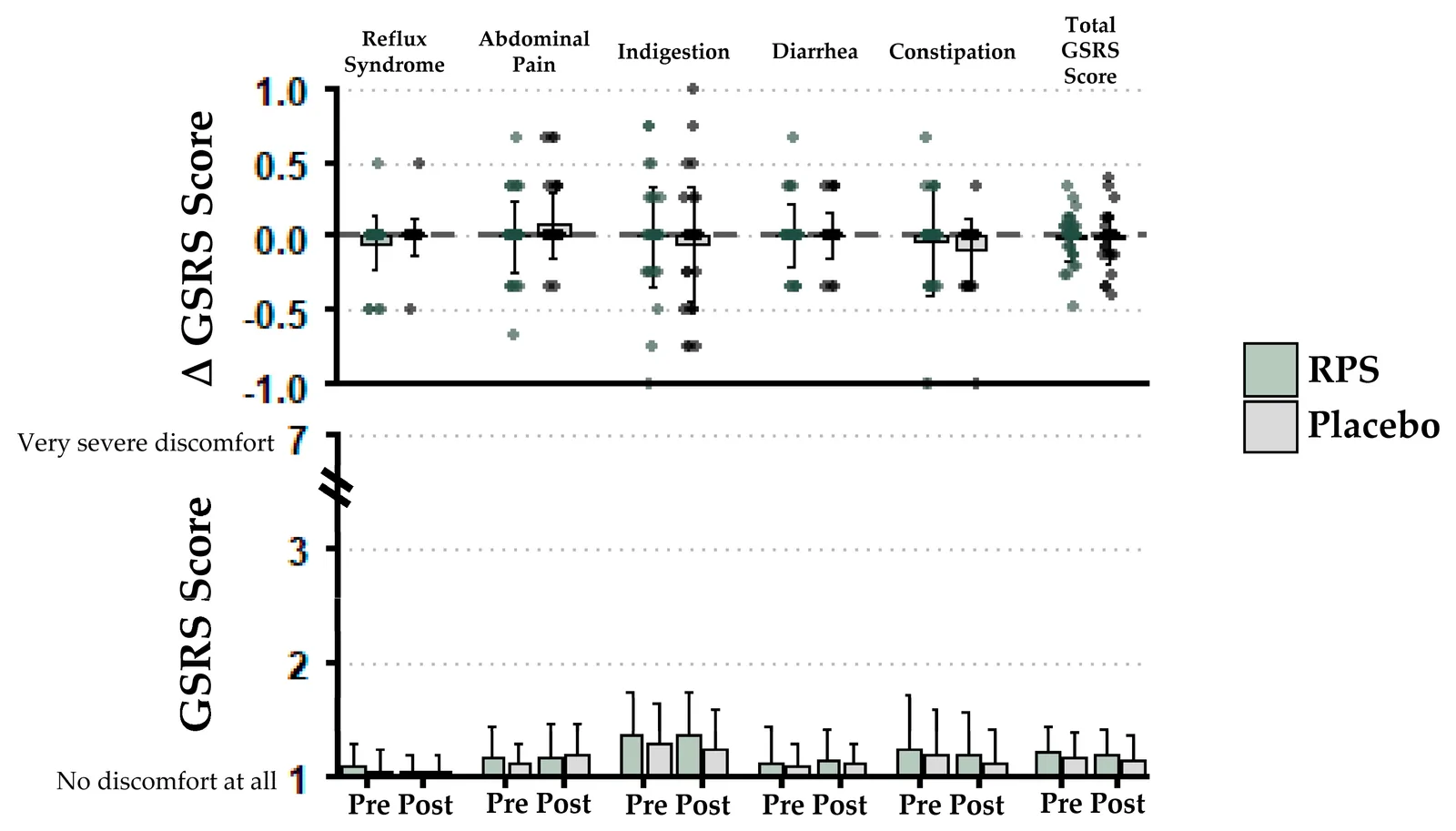
Gastrointestinal Symptom Rating Scale (GSRS) scores following resistant potato starch (RPS) supplementation. Upper plots show individual changes in GSRS scores for reflux syndrome, abdominal pain, indigestion, diarrhea, constipation, and total GSRS scores for RPS relative to placebo. Lower plots show mean GSRS scores during the last seven days of the Run-In and Treatment periods for RPS and placebo. Higher scores indicate greater gastrointestinal distress. Between- and within-treatment differences were assessed using Student’s *t*-tests with Bonferroni corrections for within-treatment comparisons. Data are shown as mean ± standard deviation.

To evaluate the gastrointestinal tolerance of RPS supplementation, participants completed the GSRS for the last 7 days of the Run-In and Treatment periods. Exploratory analysis revealed no differences in the changes in individual category and total GSRS scores between the RPS and placebo groups. There was a trend towards reduction in constipation (*t*-test; *p* = 0.054) for the placebo, but RPS had no effect on GSRS scores (Figure 2). Overall, RPS supplementation was shown to be well tolerated, with findings similar to the previous clinical trial, as daily consumption of 3.5 g RPS, 7 g RPS, or the placebo had no effect on abdominal pain, belching, bloating, gas, or feeling unwell [33].

### 3.5. Bristol Stool Form Scale (BSFS) Reporting

Contrary to the previous clinical trial [15], secondary endpoint analysis revealed no difference in the abundance of BSFS Type 1 or 2 stools (Chi-Square; *p* = 0.9) or Type 7 stools (*p* = 1.0) during the last seven bowel movements between the treatment groups in the present study (Table 3). Given that reductions in the proportion of undesirable stools should also increase the number of normal stools, the number of normal stools (BSFS Type 3 or 4) during the last seven bowel movements was compared between the RPS and placebo treatment groups. There were more normal stools in the RPS treatment group compared to the placebo group (Table 3, *p* = 0.0077). Considered in a different way, more RPS participants experienced a majority (>50%) of normal Type 3 or 4 BMs during the last seven BMs recorded compared to placebo participants (Chi-Square; *p* = 0.013). The results from the FAS population were consistent with the PP population (Supplementary Table S3).

The number of normal stools (Type 3 or 4) was not compared between the placebo and 3.5 g RPS groups in the previous clinical trial [15]. Exploratory reanalysis of these data revealed that the number of normal stools (Type 3 or 4) was higher in the 3.5 g RPS per day group versus the placebo group (Table 3, *p* = 0.012).

### 3.6. Bowel Movement Frequency

**Figure 3:**
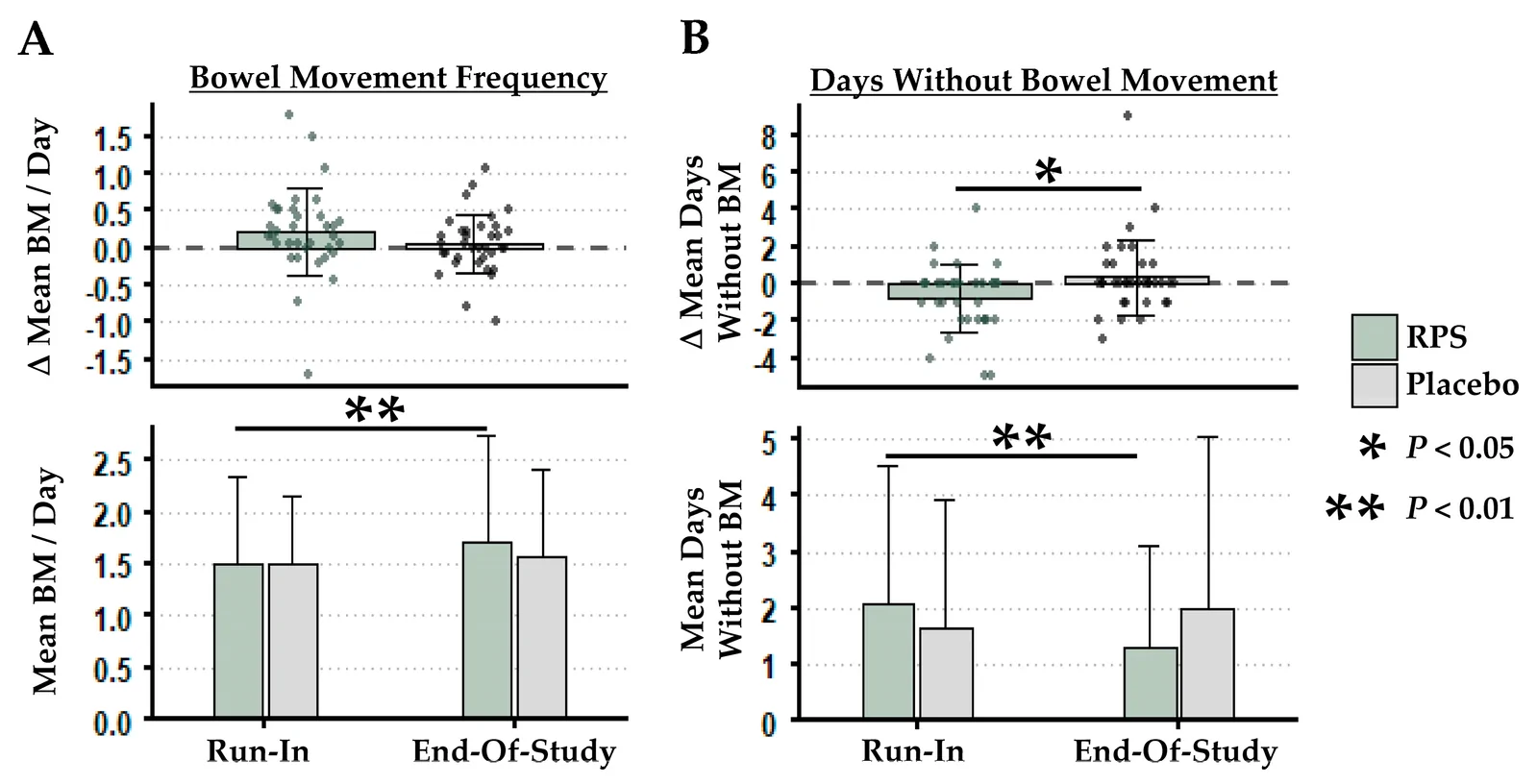
Bowel movement frequency and number of days without a bowel movement (BM) following resistant potato starch (RPS) supplementation. (**A**) Mean BM frequency for RPS and placebo over 14 days. (**B**) Mean number of days without a BM for RPS and placebo over 14 days. Upper plots show individual BM changes for RPS relative to placebo. Lower plots show BM changes during the last 14 days of Run-In and Treatment periods for RPS and placebo. Between- and within-treatment (placebo) differences were assessed using Student’s *t*-test. Within-treatment (RPS) differences were assessed using the Wilcoxon signed-rank test with Bonferroni corrections. Data are shown as mean ± standard deviation.

Five record days were missing during the Run-In and sixteen record days were missing during the End-of-Study for the placebo group, and five record days were missing during the Run-In and two record days were missing during the End-of-Study for the RPS group. Given that having a BM prompted participants to refer to the BSFS for scoring and the absence of a BM removed this prompt, it was assumed that missing record days were due to an absence of BMs on that day. The change in the number of BMs during the final 14 days of the trial compared to the 14-day Run-In period was normally distributed in the placebo group (Shapiro–Wilk test, W = 0.963, *p* = 0.3) but not in the RPS group (W = 0.922, *p* = 0.016). Exploratory analysis revealed that the average number of BMs during the final 14 days of the trial was not different compared to the 14-day Run-In period in the placebo group (Figure 3A; *t*-test, *p* = 0.88). The RPS group experienced an average of three more BMs during the final 14 days when compared to the Run-In period (Figure 3A; Wilcoxon signed-rank test, *p* < 0.0001), but this was not different from the placebo group (*t*-test, *p* = 0.18).

Further exploratory analysis revealed that the average number of days without a BM decreased by almost one full day in the RPS group over the same period (Wilcoxon signed-rank test, *p* = 0.0008), which was significantly less than the change in the placebo group (*t*-test, *p* = 0.019) (Figure 3B). The average number of days without a BM was unchanged between the Run-In period and the final 14 days in the placebo group (Figure 3B; *t*-test, *p* = 0.66). To address the possibility of scoring missing record days as days without a BM, missing participant values were replaced with imputed data based on the number of participant-specific data recorded during the Run-In and End-of-Study periods. Reanalysis of these data using imputed values led to similar conclusions (Supplementary Table S4).

### 3.7. Changes in Bifidobacterium and Akkermansia

We previously reported that consuming 3.5 g of RPS per day for 4 weeks led to significantly greater increases in *Bifidobacterium* and *Akkermansia* compared to a placebo [15]. Based on these previous findings, the present study assessed the effects of RPS supplementation on the most abundant *Akkermansia*- and *Bifidobacterium*-related OTUs and higher taxonomic levels, as determined a priori by the previous findings [15]. Relative abundances of *Bifidobacterium* (Supplementary Table S5), *Akkermansia* (Supplementary Table S6), and related taxa were log-transformed, and absolute z-scores were generated. Only one fecal sample exceeded the ±2 standard deviation threshold for *Bifidobacterium,* and the data from this participant were excluded from analysis (Supplementary Table S5). No fecal sample exceeded ±2 standard deviations for *Akkermansia* (Supplementary Table S6). Furthermore, relative abundance changes were assessed at each taxonomic level for normality using the Shapiro–Wilk test and were found to be not normal (RPS *Bifidobacterium*: W = 0.394, *p* < 0.0001; placebo *Bifidobacterium:* W = 0.702, *p* < 0.0001) (RPS *Akkermansia*: W = 0.747, *p* < 0.0001; placebo *Akkermansia*: W = 0.384, *p* < 0.0001). Relative abundance changes also tended to display heteroscedasticity. Specifically, for changes in *Bifidobacterium*, RPS variance = 9.17 and placebo variance = 0.917 (Levene’s test, *p* = 0.033), and for *Akkermansia*, RPS variance = 5.31 and placebo variance = 104 (Levene’s test; *p* = 0.069). *Bifidobacterium* was also undetected in 38 samples, and 12 participants reported zero-change values (17.4%). *Akkermansia* was undetected in 31 samples, and 10 participants reported zero-change values (14.3%). For these collective reasons, changes in relative abundances between treatment groups were compared using the Brunner–Munzel test.

**Figure 4:**
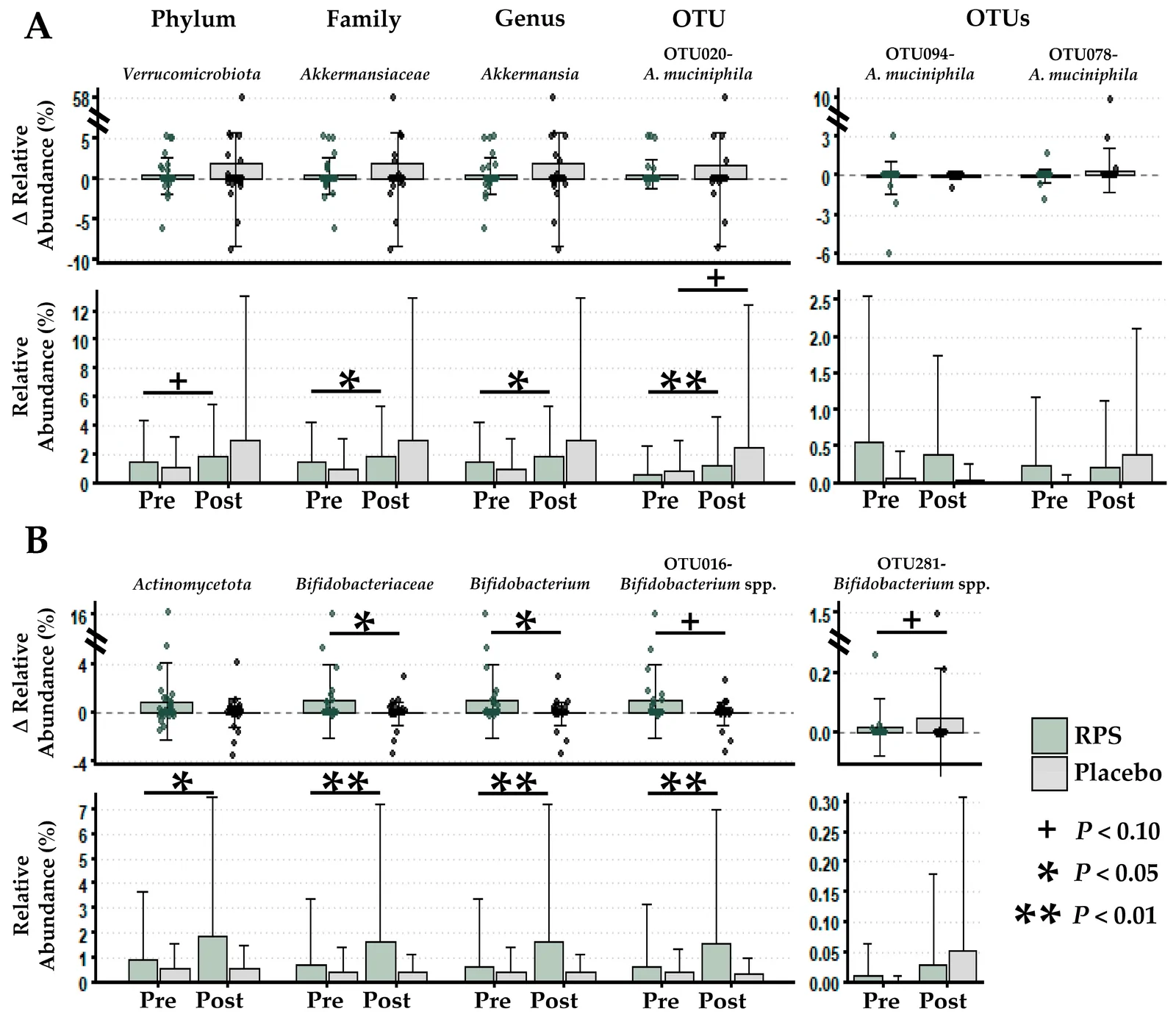
Effects of resistant potato starch (RPS) supplementation on abundant *Akkermansia*- and *Bifidobacterium*-related OTUs and higher taxonomic levels as determined *a priori*. (**A**) *Verrucomicrobiota*-, *Akkermansiaceae*-, *Akkermansia*-, and *Akkermansia*-related OTU relative abundances for RPS and placebo. (**B**) *Actinomycetota*-, *Bifidobacteriaceae*-, *Bifidobacterium*-, and *Bifidobacterium*-related OTU relative abundances for RPS and placebo. Upper plots show individual relative abundance changes for RPS relative to placebo. Lower plots show relative abundance at baseline (Pre) and End-of-Study (Post) for RPS and placebo. Between-treatment differences were assessed using the Brunner–Munzel test, as appropriate, while within-treatment differences were assessed using the Wilcoxon signed-rank test with Pratt’s signed-rank zero procedure and Bonferroni corrections. Data are shown as mean ± standard deviation. OTU, operational taxonomic unit.

To accomplish the primary research objective, which was to test whether RPS stimulated a greater increase in *Akkermansia* relative to the placebo, we compared End-of-Study changes in *Akkermansia* relative abundance between RPS and the placebo for PP populations. This analysis showed that RPS-induced changes in *Akkermansia* were no different from the placebo (Figure 4A; Brunner–Munzel test; *p* = 0.49). However, further within-group exploratory testing for RPS revealed that relative abundances of *Akkermansiaceae* (*p* = 0.046), *Akkermansia* (*p* = 0.046), and *Akkermansia muciniphila*-related OTU020 (*p* = 0.0001) were increased relative to baseline, an effect that was not observed for the placebo (Wilcoxon signed-rank test; *p* > 0.05) (Figure 4A). Additional analysis based on FAS populations further revealed no significant between-group changes in *Akkermansia* (*p* = 0.37).

To address the secondary research objective, End-of-Study changes in the relative abundance of *Bifidobacterium* were compared between RPS and the placebo for PP populations. This analysis revealed that RPS-induced changes in *Bifidobacterium* were significantly greater than the placebo (Figure 4B, *p* < 0.0001). Further exploratory analysis revealed that *Bifidobacteriaceae* relative abundance was also greater for RPS relative to the placebo (Figure 4B, *p* < 0.0001). Moreover, relative abundances of *Actinomycetota* (*p* = 0.044), *Bifidobacteriaceae* (*p* < 0.0001), *Bifidobacterium* (*p* < 0.0001), and *Bifidobacterium*-related OTU016 (*p* = 0.0001) were shown to be enriched relative to baseline with RPS but not with the placebo (*p* > 0.10) (Figure 4B). While additional analysis based on FAS populations showed no significant between-group changes in *Bifidobacterium* (*p* = 0.10), within-RPS-group differences were observed for *Bifidobacterium* when compared to baseline (*p* = 0.0004; Supplementary Table S7).

### 3.8. Reanalysis of Bifidobacterium and Akkermansia Changes in the Previous Study

Exploratory reanalysis of changes in bacterial abundance from the previous clinical trial [15] followed the same process as described above. Relative abundances of *Bifidobacterium* (Supplementary Table S8) and *Akkermansia* (Supplementary Table S9) were log-transformed, and absolute z-scores were generated, but no samples exceeded ±2 standard deviations. Changes in the relative abundance of *Bifidobacterium* and *Akkermansia* were tested for normality using the Shapiro–Wilk test and were found to be not normal (placebo *Bifidobacterium*: W = 0.646, *p* < 0.0001; 3.5 g RPS *Bifidobacterium*: W = 0.890, *p* = 0.013; placebo *Akkermansia*: W = 0.736, *p* < 0.0001; 3.5 g RPS *Akkermansia*: W = 0.873, *p* = 0.0059). Changes in relative abundance were assessed for heteroscedasticity: For changes in *Bifidobacterium*, the placebo group variance = 0.017 and the 3.5 g RPS group variance = 0.015 (*p* = 0.74; Levene’s test). For *Akkermansia*, the placebo group variance = 0.050 and the 3.5 g RPS group variance = 0.032 (*p* = 0.30; Levene’s test). *Bifidobacterium* was absent in 11 samples, and *Akkermansia* was absent in two samples. The number of absent samples was greater in the present study for both *Bifidobacterium* (Chi-Square; *p* = 0.0058) and *Akkermansia* (*p* < 0.0001). There were no zero-change values in either the *Bifidobacterium* or *Akkermansia* data sets, which was notably different from the present study for both *Bifidobacterium* (*p* = 0.0025) and *Akkermansia* (*p* = 0.0062). Due to a lack of heteroscedasticity and ties arising from zero-change values, changes in relative bacterial abundance between treatment groups were compared using the Mann–Whitney U test.

**Figure 5:**
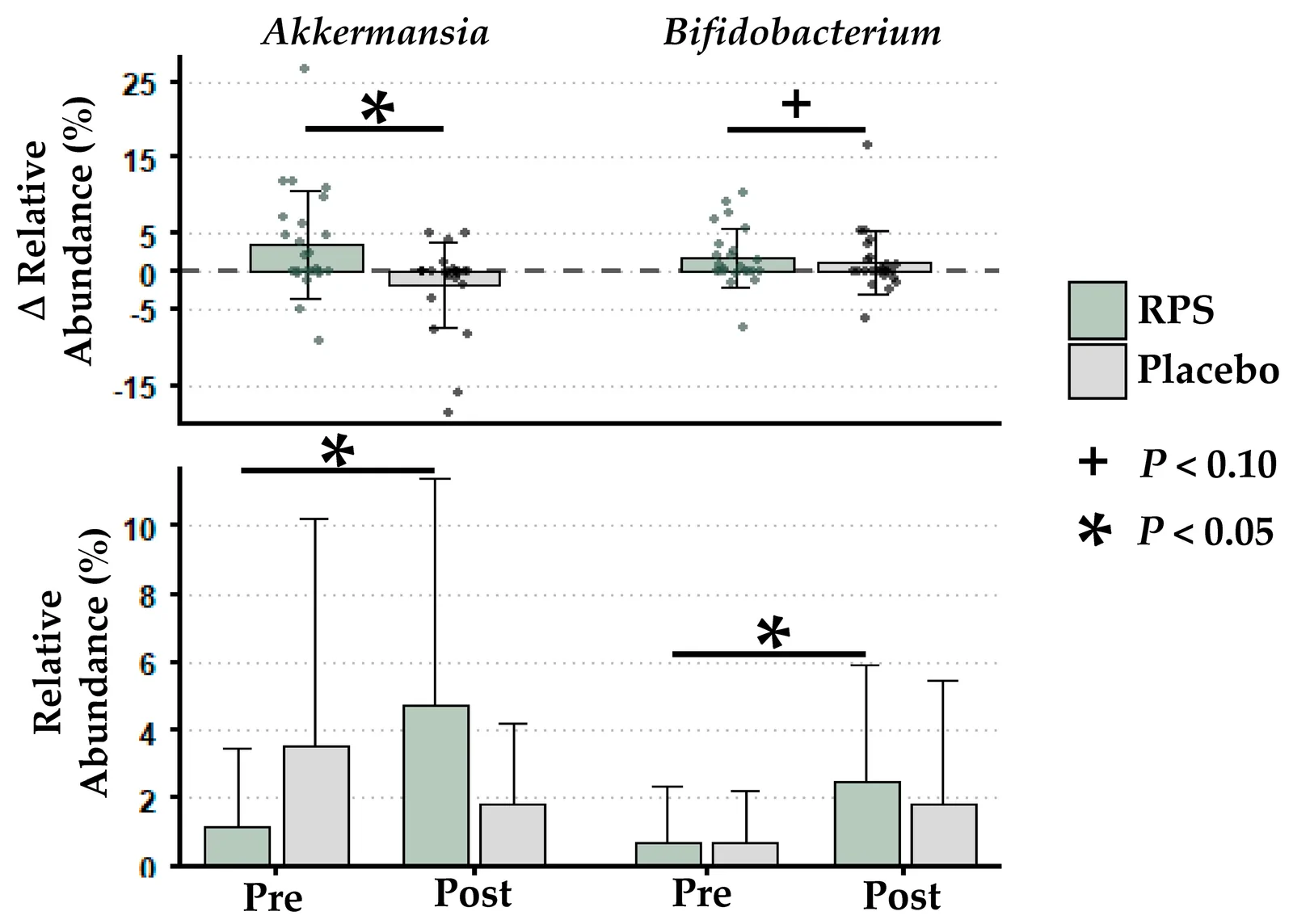
Changes in the relative abundance of *Akkermansia* and *Bifidobacterium* following resistant potato starch (RPS) supplementation. Upper plots show individual changes in the relative abundance of *Akkermansia* and *Bifidobacterium* for RPS relative to placebo. Lower plots show relative abundance at baseline (Pre) and End-of-Study (Post) for the RPS and placebo. Between-treatment differences were assessed using the Mann–Whitney U test, as appropriate, while within-treatment differences were assessed using the Wilcoxon signed-rank test with Pratt’s signed-rank zero procedure and Bonferroni corrections. Data are shown as mean ± standard deviation.

The median change in *Bifidobacterium* tended to be greater in the 3.5 g RPS per day group compared to the placebo group (Figure 5, Mann–Whitney U test, *p* = 0.10). There was an increase in *Bifidobacterium* within the 3.5 g RPS group (Figure 5, Wilcoxon signed-rank test, *p* = 0.019) but not within the placebo group (*p* = 0.44). The median Week 4 change in *Akkermansia* was greater in the 3.5 g RPS per day group compared to the placebo group (Figure 5, *p* = 0.010). Additionally, *Akkermansia* levels increased within the 3.5 g RPS per day group (Figure 5, *p* = 0.034) but not within the placebo group (*p* = 0.30).

## 4. Discussion

Prebiotics are substrates that are selectively utilized by host microorganisms to confer a health benefit [1], and reproducing these effects in clinical trials to provide confirmatory evidence is one of the key criteria stipulated by ISAPP in classifying compounds as prebiotics [2]. A previous study found that daily consumption of 3.5 g of RPS for 4 weeks led to increases in *Bifidobacterium* and *Akkermansia*, as well as fewer instances of BSFS Type 7 and Type 1 or 2 stools compared to a placebo in otherwise healthy people [15]. In the present study, which followed a similar protocol comparing 3.5 g of RPS to a fully digestible corn starch placebo over 4 weeks in otherwise healthy people, there were no serious adverse events, and both interventions were well tolerated, as evidenced by the GSRS survey results. RPS supplementation increased *Bifidobacterium* levels compared to the placebo group, meeting the secondary objective. The prespecified primary endpoint, confirming that RPS supplementation led to greater increases in *Akkermansia*, did not reach statistical significance between treatment groups. However, *Akkermansia* levels were higher at the End-of-Study period compared to the Run-In period in the RPS group but not in the placebo group. Analysis of the FAS revealed no significant differences between treatment groups for changes in either *Bifidobacterium* or *Akkermansia*, due in part to poor compliance by one participant in the RPS group and unexplained increases in both genera in poorly compliant participants in the placebo group. The abundance of constipation-associated and diarrhea-associated bowel movements, also secondary objective measures, was not different between the RPS and placebo groups. However, supplementation with RPS increased the number of normal stools compared to the placebo group, an effect noted in both the PP and FAS populations. Bowel movement frequency was not different between groups, but participants consuming RPS had more BMs after 4 weeks of supplementation compared to the Run-In period. Furthermore, participants in the RPS group had fewer days without a BM compared to those consuming the placebo during the final 14 days of the trial. Applying the same statistical approaches used here to the 3.5 g and placebo microbiota and stool form data from the previous clinical trial revealed similar conclusions to the present study, supporting reproducible bifidogenic effects and stool normalization at the daily RPS dose of 3.5 g.

Meta-analyses of clinical trials have reported that RS supplementation increases fecal wet weight and butyrate concentrations and reduces fecal pH, markers of metabolic syndrome, oxidative stress and inflammation, appetite and body weight [34,35,36,37,38,39,40], while others found no effect [41,42]. Benefits have also been documented in patients with chronic kidney disease [42,43], motivating ongoing research in this area [44]. Despite the physiological benefits of RS supplementation, the connection between these health improvements and changes in gut microbiota composition is poorly understood. Besides RPS [15], other indigestible carbohydrates have demonstrated prebiotic effects, substantiated by increases in bifidobacteria and improvements in stool form. Trans-galactooligosaccharides [45] and pectin [46] improved stool form and increased *Bifidobacterium* in patients with IBS. Partially hydrolyzed guar gum reduced BSFS scores and increased *Bifidobacterium* levels in people tending to experience diarrhea (50% of BMs Type 5 or 6) [47] and reduced constipation scores while increasing *Bifidobacterium* in hemodialysis patients [48]. However, differences in doses, study durations, and populations complicate reproducibility comparisons among these prebiotics.

Although few studies have aimed to explicitly reproduce the effects of prebiotics in humans, there is one exceptional example. Compared to a fully digestible corn starch placebo, daily consumption of a high (40 g/day) dose of high-amylose maize starch (HAMS; an RS Type 2) decreased intrahepatic triglyceride levels, body weight, and liver enzymes in patients with metabolic dysfunction-associated steatotic liver disease (MASLD) [49]. These effects were connected to a reduction in *Bacteroides stercoris*, a species of bacteria previously connected to MASLD symptoms in human cohorts [50,51]. A follow-up multicenter, single-arm study evaluating the same dose of HAMS for the same duration found remarkably similar metabolic effects [52]. However, responder–nonresponder analysis did not connect *Bacteroides stercois* changes to symptomatic improvement. Rather, *Prevotella copri* and *Bifidobacterium pseudocatenulatum* were found to negatively and positively mediate the beneficial effects, respectively, of HAMS on MASLD symptoms [52]. These findings suggest that heterogeneous changes in the gut microbiota can contribute to similar phenotypic changes in humans in response to RS. In this context, the effects of RPS are remarkably similar despite modest differences in relative abundance changes and stool form changes between studies.

Changes in *Bifidobacterium* and normalization of stool form scores showed the greatest agreement between trials. *Bifidobacterium* is known to utilize RS, and levels of this genus increase in response to RPS supplementation [10,11,12]. Conversely, *Akkermansia* is not a primary degrader of RS and feeds primarily on host mucins [53,54]. The primary objective of this study was to demonstrate that RPS produced significantly greater increases in *Akkermansia* compared to the placebo, which was not met. It is interesting to note that RPS supplementation elicited a within-group *Akkermansia* increase and that, while insignificant, *Akkermansia* levels tended to increase in the placebo group. Dietary substances like RS Type 2 increase the abundance of *Akkermansia* [15,55,56,57], suggesting that depolymerization of starch into monomeric glucose may act as a substrate for this genus. However, there is limited in vitro evidence linking increased abundance of this genus to glucose utilization [53], suggesting *Akkermansia* increases in the presence of RS may occur via microbial ecological interactions and potentially via interactions between the microbiota and the host. Further studies examining a more complete representation of the gut microbiome, including strain-level characterization, could improve our understanding of the bifidogenic response to RPS.

The frequencies of constipation- (Type 1 or 2) and diarrhea-associated (Type 7) stools at 4 weeks were lower in the 3.5 g RPS group in the previous study [15] but were similar in the RPS and placebo groups in the present study. The stool form differences reported in the previous study were driven by a lower incidence of Type 2 stools in the 3.5 g RPS group and a higher incidence of Type 7 stools in the placebo group during the last seven recorded BMs [15]. However, there were more normal stools (Type 3 or 4) during the last seven recorded BMs in the RPS group in both studies. Increases in Type 3 or 4 stools are generally regarded as the target stool types in both healthy [27,58] and unhealthy [58,59,60] populations. Few dietary fibers have an ambilateral effect on stool form by providing benefit to people suffering from both constipation and diarrhea. Partially hydrolyzed guar gum improves stool form in healthy participants with a tendency towards diarrhea [47] but has mixed effects in constipated patients [61,62,63]. Psyllium husk from certain species of *Plantago* mechanically achieves stool form normalization due to high mucilage content, which binds water to reduce diarrhea [64] and increases bulk to relieve constipation [65]. Notably, psyllium is not appreciably fermented by gut microbiota [66], consistent with these benefits arising from the water-binding effects of the mucilaginous content. Unlike psyllium, RPS does not gel or otherwise interact with water in the digestive tract; rather, stool form normalization appears to be due to changes in various taxa [33].

Stool form analysis was separated from BM frequency by characterizing the last seven BMs for each participant, and there was a higher proportion of normal stools in the RPS treatment compared to the placebo treatment in both the past and present study. In the present study, there were fewer days without a BM in the RPS group compared to the placebo group, and while BM frequency was not different between the groups, RPS produced a within-group increase in BM frequency. Increased BM frequency can be associated with diarrhea [58], which can be a side effect of some prebiotics [67]. On the contrary, within-group increases in BM frequency were due, in part, to an increase in the number of days with a BM, a validated marker of constipation relief [68]. Increasing BM frequency and normalizing stool form while reducing days without a BM without increasing GSRS symptoms suggests that RPS simultaneously promotes multiple aspects of BM normalization. The gastrointestinal benefits of RPS described here occurred in healthy adults, so any clinical relevance in diseased populations remains to be determined. Bifidogenic increases and normalization of stool form, combined with good tolerability, make RPS an interesting candidate for studies evaluating prebiotic efficacy in patient populations, including those with functional constipation and IBS.

There are several notable strengths and limitations of this clinical trial. The present study was conducted consistently with the previous study, following identical RPS and placebo dosages, double-blind methodology, randomization and allocation strategies, target study duration, and selection of an otherwise healthy population from the same geographic region. Targeted analysis of beneficial bacteria and the use of the BSFS are also strengths because these are validated measurements valued by dietary supplement and food regulatory agencies. Prebiotic substantiation, as defined by ISAPP, suggests confirmatory evidence can increase confidence in prebiotic ingredients [2], which contributes to regulatory certainty for products containing these ingredients. However, previous efforts to replicate the effects of prebiotic ingredients have only been partially successful [4,5,6]. The use of different microbiome extraction techniques and sequencing methods in the present study likely contributed to the blunted bifidogenic effect and lack of *Akkermansia* response between treatment groups when compared to the previous study. Consumption of RS from naturally occurring dietary sources may also influence these responses, but macronutrient intake was not affected, and RS levels in food are strongly influenced by cooking methods and storage conditions [69], making it difficult to accurately determine nutritional intake of this dietary fiber [70,71]. Changes in *Akkermansia* were comparable between treatment groups in the present study. It is possible that the power calculation used to determine the study sample size may have overestimated the expected effect of RPS on *Akkermansia* and that an insufficient number of participants enrolled in this study contributed to the lack of effect. Future studies aimed at understanding how baseline microbiota composition and other physiological factors influence the positive *Bifidobacterium* and *Akkermansia* responses to RPS supplementation are warranted.

## 5. Conclusions

Daily supplementation with 3.5 g of RPS for four weeks consistently led to increases in *Bifidobacterium* and normalized stool form in otherwise healthy people in two independent clinical studies. Supplementation also led to increases in BM frequency and reduced days without a BM without affecting GSRS scores in the present study. Collectively, these results substantiate the prebiotic benefits and tolerability of supplementing with RPS.

## Figures and Tables

**Table 1:** Baseline demographic data for the full analysis set (FAS) population.

	Placebo	RPS	Total
Number of Participants	37	36	73
Age (Year) ^1^			
Mean (SD)	41.9 (14.3)	41.9 (16.6)	41.9 (15.4)
Median (Min–Max)	45 (19, 64)	38 (18, 69)	41 (18, 69)
Sex			
Female	17 (45.9%)	20 (55.6%)	37 (50.7%)
Male	20 (54.1%)	16 (44.4%)	36 (49.3%)
Race			
Asian Indian	2 (5.4%)	3 (8.3%)	5 (6.8%)
Black or African American	2 (5.4%)	4 (11.1%)	6 (8.2%)
Chinese	0 (0%)	2 (5.6%)	2 (2.7%)
Filipino	3 (8.1%)	2 (5.6%)	5 (6.8%)
Not Reported	6 (16.2%)	7 (19.4%)	13 (17.8%)
Other Asian	1 (2.7%)	1 (2.8%)	2 (2.7%)
White	21 (56.8%)	17 (47.2%)	38 (52.1%)
White, Asian Indian	1 (2.7%)	0 (0%)	1 (1.4%)
White, Japanese	1 (2.7%)	0 (0%)	1 (1.4%)
Ethnicity			
Hispanic or Latino	4 (10.8%)	3 (8.3%)	7 (9.6%)
Not Hispanic or Latino	33 (89.2%)	33 (91.7%)	66 (90.4%)
Height (cm)			
Mean (SD)	169.1 (10.5)	167.9 (9.1)	168.5 (9.8)
Median (Min–Max)	170.6 (149.3–193.0)	166.5 (143.8–187.2)	168.3 (143.8–193.0)
Weight (kg)			
Mean (SD)	75.2 (15.4)	71.9 (11.1)	73.6 (13.5)
Median (Min–Max)	74.7 (47.7–104.9)	72.0 (56.7–95.1)	72.5 (47.7–104.9)
BMI (kg/m^2^)			
Mean (SD)	26.1 (3.7)	25.5 (3.4)	25.8 (3.5)
Median (Min–Max)	26.9 (20.2–33.2)	24.9 (19.4–34.1)	25.5 (19.4–34.1)

Age, sex, race, ethnicity, height, weight, and body mass index (BMI) for full analysis set (FAS) participants at baseline. ^1^ Age calculated based on the date of informed consent signed and date of birth. Resistant potato starch (RPS). Median, and minimum and maximum (Min-Max) are indicate where applicable. Standard deviation (SD) is indicated where applicable.

**Table 2:** Summary of treatment-emergent adverse events by treatment groups.

	Placebo		RPS		Total	
	Participants (n = 37)	Events (n = 9)	Participants (n = 36)	Events (n = 9)	Participants (n = 73)	Events (n = 18)
**Overall**	7	9	5	9	13	18
Serious	0	0	0	0	0	0
Fatal	0	0	0	0	0	0
Discontinuation	0	0	0	0	0	0
**Severity**						
Mild	7	9	5	8	13	17
Moderate	0	0	1	1	1	1
Severe	0	0	0	0	0	0
**Relationship**						
Related	0	0	0	0	0	0
Possibly Related	2	2	0	0	2	2
Unlikely Related	1	1	2	2	3	3
Not Related	5	6	4	7	9	13

Treatment-emergent adverse events (TEAEs) reported in the full analysis set population were neither serious nor fatal, and no TEAEs led to study discontinuation. Participant numbers in the Severity and Relationship columns exceed the number in the Overall column because some participants experienced multiple events with discrete severity and/or relationship to the interventions. The majority of TEAEs were determined to be unrelated to the interventions, and no TEAEs were definitively related to the interventions. Resistant potato starch (RPS).

**Table 3:** Bristol Stool Form Scale scores for the last seven recorded bowel movements reported for the present and previous clinical trials.

	Present Clinical Trial							
	Type 1	Type 2	Type 3	Type 4	Type 5	Type 6	Type 7	Total
RPS (3.5 g)	11	22	40	119	27	24	2	245
Placebo	10	24	19	111	54	25	2	245
	**Previous Clinical Trial** [15]							
RPS (3.5 g)	2	7	36	83	22	18	0	168
Placebo	3	20	23	73	36	7	6	168

## Data Availability

The data that support the findings of this study are available from MSP Starch Products Inc., but restrictions apply to the availability of these data, which were used under license for the current study and so are not publicly available. Data are, however, available from the authors upon reasonable request and with permission from MSP Starch Products Inc. Individual patient data are unavailable as per the informed consent form and the interventional study protocol registration.

## Supplementary Materials

The following supporting information can be downloaded at https://www.mdpi.com/article/10.3390/nu18213538/s1: Table S1. Bristol Stool Form Scale scores for bowel movements recorded during the Run-In period for participants allocated to the placebo and RPS groups; Table S2. Changes in macronutrient intake between Visit 2 and Visit 3; Table S3. The last seven BSFS Scores from the FAS population were compared between treatment groups using Chi Square analysis; Table S4. Recalculation of BM Frequency and Days with No BM during the 14-Day Run-In and 14-Day End-of-Study Periods using Imputed Values; Table S5. Z-scores derived from *Bifidobacterium* relative abundance values; Table S6. Z-scores derived from *Akkermansia* relative abundance values; Table S7. Changes in Bifidobacterium and Akkermansia in the FAS population; Table S8. Z-scores derived from *Bifidobacterium* relative abundance values from the 2023 trial; Table S9. Z-scores derived from *Akkermansia* relative abundance values from the 2023 trial.

## Abbreviations

The following abbreviations are used in this manuscript:

AE	Adverse Event
BM	Bowel Movement
BMI	Body Mass Index
BSFS	Bristol Stool Form Scale
FAS	Full Analysis Set
GI	Gastrointestinal
GSRS	Gastrointestinal Symptom Rating Scale
HAMS	High-Amylose Maize Starch
IBS	Irritable Bowel Syndrome
ICF	Informed Consent Form
ICH	International Council for Harmonization
IRB	Institutional Review Board
ISAPP	International Associate of Probiotics and Prebiotics
OTU	Operational Taxonomic Unit
MASLD	Metabolic Dysfunction-Associated Steatotic Liver Disease
PP	Per-Protocol Population
RS	Resistant Starch
RPS	Resistant Potato Starch
SAF	Safety Analysis Set
SD	Standard Deviation
TEAE	Treatment-Emergent Adverse Event
