# Supplementary material for: Examining the Gastrointestinal Effects of Solnul®, a Prebiotic Resistant Potato Starch: A Replication Randomized, Double-Blind, Placebo-Controlled Clinical Trial in Healthy Adults
Source: Nutrients. 2026 Oct 27;18(21):3538. doi: 10.3390/nu18213538 (PMC13765139; doi:10.3390/nu18213538)
Supplement: Supplementary file 1 [file nutrients-18-03538-s001.zip › nutrients-4620546-supplementary.pdf]

Supplementary Materials

Supplementary Table S1

Supplementary Table S1. Bristol Stool Form Scale scores for bowel movements recorded during the Run-In period for participants allocated to the placebo and RPS groups.

	Type 1	Type 2	Type 3	Type 4	Type 5	Type 6	Type 7	Total
**RPS**	25 (3%)	41 (6%)	107 (14%)	326 (44%)	170 (23%)	60 (8%)	11 (1%)	740
**Placebo**	24 (3%)	83 (11%)	158 (21%)	312 (41%)	125 (17%)	48 (6%)	7 (1%)	757

Supplementary Table S2

Supplementary Table S2. Changes in macronutrient intake between Visit 2 and Visit 3.

Mean (SD)	Calories (kcal)	Carbohydrates (g)	Protein (g)	Fat (g)
**RPS**	-7.4 (689)	-13.1 (80)	-3.2 (47)	4.8 (39)
**Placebo**	80 (510)	15 (54)	7.6 (27)	-0.4 (32)
p value	0.54	0.089	0.24	0.54

Changes were compared using Student's *t*-test.

Supplementary Table S3

Supplementary Table S3. The last seven BSFS Scores from the FAS population were compared between treatment groups using Chi Square analysis.

	Type 1	Type 2	Type 3	Type 4	Type 5	Type 6	Type 7	Total
RPS	11	22	40	120	29	25	4	251
Placebo	10	25	20	121	55	26	2	259
p value	0.9		0.033				0.39	

Supplementary Table S4

Supplementary Table S4. Recalculation of BM Frequency and Days with No BM during the 14-Day Run-In and 14-Day End-of-Study Periods using Imputed Values.

BM Frequency (BM/Day)

	Run-In	End-of-Study	Change	Between Group *p* value	Within-Group *p* value
Placebo	1.52 (0.66)	1.6 (0.83)	0.078 (0.39)	0.3	0.22
RPS	1.52 (0.84)	1.72 (1.02)	0.2 (0.58)		0.015

Days with No BM (Days)

	Run-In	End-of-Study	Change	Between Group *p* value	Within-Group *p* value
Placebo	1.49 (2.16)	1.6 (2.86)	0.11 (1.45)	0.039	0.034
RPS	1.94 (2.49)	1.26 (1.74)	-0.69 (1.71)		0.0005

Between group *p*-values are from *t*-test comparisons and within group *p*-values are from Wilcoxon Signed-Rank tests with Bonferroni corrections. Values are presented as Mean (SD).

Supplementary Table S5. Z-scores derived from *Bifidobacterium* relative abundance values. Values with a Z-score greater than two are considered outliers and are indicated in bold.

Subject ID	Timepoint	Relative Abundance (%)	Log10(x+0.0001)	Z-Score (Absolute value of ((x-Mean)/Standard Deviation))
1003	Baseline	0.035917	-1.443	0.164
1005	Baseline	0.015576	-1.804778174	0.060
1007	Baseline	0.04585	-1.337718532	0.230
1009	Baseline	0.636555	-0.196095879	0.938
1011	Baseline	0	-4	1.422
1013	Baseline	0.572343	-0.242267817	0.910
1016	Baseline	0	-4	1.422
1017	Baseline	0.0052	-2.275729244	0.352
1020	Baseline	0.007165	-2.138772541	0.267
1022	Baseline	16.01768	1.204602299	1.808
1024	Baseline	0.002689	-2.554511559	0.525
1025	Baseline	0.004491	-2.338097368	0.391
1027	Baseline	0	-4	1.422
1030	Baseline	0.334087	-0.476010451	0.765
1032	Baseline	0.010394	-1.979050662	0.168
1034	Baseline	0.741847	-0.129626879	0.980
1035	Baseline	1.435461	0.157021584	1.158
1038	Baseline	0.005908	-2.221264985	0.318
1039	Baseline	0.036017	-1.442287531	0.165
1041	Baseline	0	-4	1.422
1044	Baseline	0.248572	-0.60437395	0.685
1045	Baseline	0	-4	1.422
1047	Baseline	0.141611	-0.848597333	0.533
1050	Baseline	0.093659	-1.027987641	0.422
1051	Baseline	0	-4	1.422
1053	Baseline	0	-4	1.422
1056	Baseline	0.550842	-0.258894177	0.899
1057	Baseline	0.008095	-2.086440467	0.235
1060	Baseline	0.216132	-0.665079834	0.647
1061	Baseline	0	-4	1.422
1063	Baseline	0.024778	-1.604186562	0.064
1066	Baseline	0.088205	-1.054016501	0.406
1067	Baseline	0	-4	1.422
1070	Baseline	0.023621	-1.624860216	0.052
1072	Baseline	0.021217	-1.67126459	0.023
1074	Baseline	2.278716	0.357709277	1.282
1003	Week 4	3.672677	0.564994541	1.411
1005	Week 4	0.024117	-1.61587416	0.057
1007	Week 4	0.619922	-0.207592872	0.931
1009	Week 4	0.376149	-0.424524843	0.797
1011	Week 4	0.004927	-2.298663297	0.367
1013	Week 4	0.711699	-0.147642904	0.968

1016	Week 4	0	-4	1.422
1017	Week 4	0.051772	-1.285069149	0.263
1020	Week 4	0.012272	-1.907561973	0.124
1022	Week 4	32.31045	1.509344362	1.997
1024	Week 4	0	-4	1.422
1025	Week 4	0.042121	-1.374469171	0.207
1027	Week 4	0	-4	1.422
1030	Week 4	0.095743	-1.018441215	0.428
1032	Week 4	0.02133	-1.668971756	0.024
1034	Week 4	8.331378	0.920722032	1.632
1035	Week 4	2.216625	0.345711758	1.275
1038	Week 4	0	-4	1.422
1039	Week 4	0.044161	-1.353981279	0.220
1041	Week 4	0	-4	1.422
1044	Week 4	0.363032	-0.439935511	0.787
1045	Week 4	0	-4	1.422
1047	Week 4	1.904112	0.279715403	1.234
1050	Week 4	0.019872	-1.699582211	0.005
1051	Week 4	0.004201	-2.366415299	0.409
1053	Week 4	0	-4	1.422
1056	Week 4	1.920753	0.283494228	1.236
1057	Week 4	0	-4	1.422
1060	Week 4	1.274811	0.105479969	1.126
1061	Week 4	0	-4	1.422
1063	Week 4	0.030006	-1.521346925	0.116
1066	Week 4	1.577713	0.198055423	1.183
1067	Week 4	0.001598	-2.770080453	0.659
1070	Week 4	0.088601	-1.05207289	0.407
1072	Week 4	0.038633	-1.411919014	0.184
1074	Week 4	3.217125	0.507481383	1.375
1001	Baseline	4.637108	0.666256592	1.474
1004	Baseline	0.113815	-0.943420013	0.475
1006	Baseline	0.619416	-0.207947441	0.931
1008	Baseline	0.187221	-0.727412847	0.609
1010	Baseline	0	-4	1.422
1012	Baseline	0	-4	1.422
1014	Baseline	0	-4	1.422
1015	Baseline	0.013323	-1.87214451	0.102
1018	Baseline	1.266637	0.102686517	1.124
1019	Baseline	0.614562	-0.211363311	0.929
1021	Baseline	0.030789	-1.510192909	0.123
1023	Baseline	0.16679	-0.777569126	0.578
1026	Baseline	0.259911	-0.585007995	0.697
1028	Baseline	0.283408	-0.54743439	0.720
1029	Baseline	0.005478	-2.253525286	0.338
1031	Baseline	0	-4	1.422
1033	Baseline	0.005955	-2.217870305	0.316

1036	Baseline	0.226142	-0.645426736	0.660
1037	Baseline	0	-4	1.422
1040	Baseline	0.006216	-2.199544167	0.305
1042	Baseline	0.005943	-2.218772316	0.317
1043	Baseline	0.015688	-1.801663935	0.058
1046	Baseline	0.012022	-1.916408451	0.129
1048	Baseline	0	-4	1.422
1049	Baseline	2.893938	0.46150427	1.347
1052	Baseline	0.105302	-0.977152334	0.454
1054	Baseline	0.514852	-0.288232842	0.881
1055	Baseline	0	-4	1.422
1058	Baseline	0	-4	1.422
1059	Baseline	0	-4	1.422
1062	Baseline	0	-4	1.422
1064	Baseline	0.014042	-1.849489611	0.088
1065	Baseline	0	-4	1.422
1068	Baseline	0.611871	-0.213269378	0.928
1069	Baseline	2.469136	0.392562565	1.304
1071	Baseline	0.032094	-1.492224353	0.134
1073	Baseline	0.387156	-0.412002355	0.804
1001	Week 4	1.333933	0.125166525	1.138
1004	Week 4	0.241552	-0.616810498	0.677
1006	Week 4	1.554211	0.19153784	1.179
1008	Week 4	0.6642	-0.177635707	0.950
1010	Week 4	0.051318	-1.288885344	0.260
1012	Week 4	0.10406	-0.98229787	0.450
1014	Week 4	0.002194	-2.639344144	0.578
1015	Week 4	0.447151	-0.349449064	0.843
1018	Week 4	1.577985	0.198130394	1.183
1019	Week 4	36.00863	1.55640777	2.026
1021	Week 4	0.105488	-0.976386871	0.454
1023	Week 4	1.060044	0.025364825	1.076
1026	Week 4	0.074121	-1.129470824	0.359
1028	Week 4	0.799546	-0.097102265	1.000
1029	Week 4	0	-4	1.422
1031	Week 4	0	-4	1.422
1033	Week 4	0	-4	1.422
1036	Week 4	0.015931	-1.795051771	0.054
1037	Week 4	0	-4	1.422
1040	Week 4	0.0128	-1.8893965	0.113
1042	Week 4	0.016042	-1.792053963	0.052
1043	Week 4	0.00834	-2.073654893	0.227
1046	Week 4	0.006031	-2.212468731	0.313
1048	Week 4	0	-4	1.422
1049	Week 4	0.515007	-0.288102578	0.881
1052	Week 4	0	-4	1.422
1054	Week 4	3.432183	0.535583063	1.392

1055	Week 4	0.017031	-1.766224945	0.036
1058	Week 4	0	-4	1.422
1059	Week 4	0	-4	1.422
1062	Week 4	0.028175	-1.5485979	0.099
1064	Week 4	0.007704	-2.107690336	0.248
1065	Week 4	0	-4	1.422
1068	Week 4	0.473678	-0.324424832	0.859
1069	Week 4	0.851762	-0.069630687	1.017
1071	Week 4	0.04199	-1.375823607	0.206
1073	Week 4	0.76618	-0.115612556	0.988

Supplementary Table S6

Supplementary Table S6. Z-scores derived from *Akkermansia* relative abundance values. Values with a Z-score greater than two are considered outliers and are indicated in bold

Subject ID	Timepoint	Relative Abundance (%)	Log10(x+0.0001)	Z-Score (Absolute value of ((x-Mean)/Standard Deviation))
1003	Baseline	0.004144276	-2.372196376	0.470
1005	Baseline	11.62971808	1.065572921	1.581
1007	Baseline	0.891982577	-0.049594943	0.916
1009	Baseline	1.415726038	0.151009895	1.035
1011	Baseline	3.404277436	0.532037703	1.263
1013	Baseline	0.0246169	-1.607006008	0.014
1016	Baseline	0.008336807	-2.073821886	0.292
1017	Baseline	0.005199938	-2.275729244	0.413
1020	Baseline	0.218528337	-0.660293549	0.551
1022	Baseline	0	-4	1.442
1024	Baseline	0	-4	1.442
1025	Baseline	7.315758746	0.864265312	1.461
1027	Baseline	0	-4	1.442
1030	Baseline	0	-4	1.442
1032	Baseline	0.0259855	-1.583600833	0.000
1034	Baseline	0	-4	1.442
1035	Baseline	0.233592043	-0.631356075	0.568
1038	Baseline	0	-4	1.442
1039	Baseline	0.005541087	-2.24863719	0.397
1041	Baseline	1.717724289	0.234978739	1.085
1044	Baseline	0	-4	1.442
1045	Baseline	4.568071459	0.659742396	1.339
1047	Baseline	0.022840437	-1.639398319	0.033
1050	Baseline	0.017028885	-1.7662709	0.109
1051	Baseline	2.949146134	0.469711019	1.225
1053	Baseline	9.664551224	0.985186186	1.533
1056	Baseline	0.023495627	-1.627168474	0.026
1057	Baseline	0	-4	1.442
1060	Baseline	0.253594997	-0.595688097	0.590
1061	Baseline	0	-4	1.442

1063	Baseline	0	-4	1.442
1066	Baseline	4.494427071	0.652684001	1.335
1067	Baseline	0.001671151	-2.751744431	0.697
1070	Baseline	0.008589589	-2.061000743	0.285
1072	Baseline	0.004243492	-2.36216102	0.464
1074	Baseline	0.006273998	-2.195588074	0.365
1003	Week 4	0.008321775	-2.074596349	0.293
1005	Week 4	17.76480803	1.249562963	1.691
1007	Week 4	0.133846804	-0.873067644	0.424
1009	Week 4	2.716424548	0.434013634	1.204
1011	Week 4	4.998768169	0.698871684	1.362
1013	Week 4	0.05083561	-1.292978485	0.174
1016	Week 4	0.026528717	-1.574649752	0.006
1017	Week 4	0.001917472	-2.695192483	0.663
1020	Week 4	3.423873026	0.534530335	1.264
1022	Week 4	0.005122951	-2.282084064	0.417
1024	Week 4	0	-4	1.442
1025	Week 4	1.103576092	0.042841635	0.971
1027	Week 4	0	-4	1.442
1030	Week 4	0	-4	1.442
1032	Week 4	0	-4	1.442
1034	Week 4	0.00782289	-2.101116386	0.309
1035	Week 4	1.708266545	0.232581058	1.084
1038	Week 4	0	-4	1.442
1039	Week 4	0.032116906	-1.491916176	0.055
1041	Week 4	9.174560217	0.962589989	1.520
1044	Week 4	0.05248655	-1.279125318	0.182
1045	Week 4	3.835711397	0.583857245	1.294
1047	Week 4	0.057700378	-1.238069324	0.206
1050	Week 4	0.049679567	-1.302948887	0.168
1051	Week 4	1.978742175	0.296411158	1.122
1053	Week 4	7.416699679	0.870216549	1.464
1056	Week 4	0.003097989	-2.495122979	0.544
1057	Week 4	0	-4	1.442
1060	Week 4	5.406765446	0.732945562	1.383
1061	Week 4	0	-4	1.442
1063	Week 4	0	-4	1.442
1066	Week 4	2.551319648	0.406781896	1.188
1067	Week 4	0.004793787	-2.310354915	0.433
1070	Week 4	0.006040958	-2.211763895	0.375
1072	Week 4	0.008915305	-2.045019595	0.275
1074	Week 4	0	-4	1.442
1001	Baseline	0.005648122	-2.240474023	0.392
1004	Baseline	7.484301413	0.874157072	1.467
1006	Baseline	0.002302662	-2.619307342	0.618
1008	Baseline	1.140347891	0.057075446	0.979
1010	Baseline	0.005771673	-2.231238166	0.386

1012	Baseline	0.007328871	-2.129077191	0.325
1014	Baseline	0.004788584	-2.310816917	0.434
1015	Baseline	0.005709935	-2.235828705	0.389
1018	Baseline	0.006031606	-2.212425787	0.375
1019	Baseline	0.133600534	-0.873866857	0.424
1021	Baseline	0	-4	1.442
1023	Baseline	0.194588584	-0.710659513	0.521
1026	Baseline	0	-4	1.442
1028	Baseline	0	-4	1.442
1029	Baseline	0	-4	1.442
1031	Baseline	2.783114388	0.444546661	1.210
1033	Baseline	0.40495474	-0.392486281	0.711
1036	Baseline	0.003015227	-2.506510316	0.551
1037	Baseline	0.484605087	-0.314522422	0.757
1040	Baseline	0	-4	1.442
1042	Baseline	0	-4	1.442
1043	Baseline	0.007844163	-2.099951873	0.308
1046	Baseline	0.165309128	-0.781440528	0.479
1048	Baseline	0.021612276	-1.663294654	0.047
1049	Baseline	0.074013767	-1.130101115	0.271
1052	Baseline	0.054940024	-1.259321387	0.194
1054	Baseline	0.020190294	-1.69271167	0.065
1055	Baseline	5.905205905	0.7712424	1.405
1058	Baseline	3.17938717	0.502357077	1.245
1059	Baseline	2.232009555	0.348715507	1.153
1062	Baseline	0.175556359	-0.755336124	0.494
1064	Baseline	0.007020993	-2.147459455	0.336
1065	Baseline	0	-4	1.442
1068	Baseline	0.003986128	-2.388688004	0.480
1069	Baseline	8.702064897	0.939627308	1.506
1071	Baseline	0	-4	1.442
1073	Baseline	0.159416989	-0.797193056	0.469
1001	Week 4	0.008326672	-2.07434391	0.293
1004	Week 4	14.97154802	1.175269608	1.647
1006	Week 4	0.013754077	-1.8584224	0.164
1008	Week 4	0.566130255	-0.247006929	0.798
1010	Week 4	0.013995801	-1.850910232	0.159
1012	Week 4	0.019270422	-1.712860922	0.077
1014	Week 4	0.00438866	-2.347883318	0.456
1015	Week 4	0.011892303	-1.921097396	0.201
1018	Week 4	0.007313951	-2.129950267	0.326
1019	Week 4	7.496487043	0.874863588	1.467
1021	Week 4	0	-4	1.442
1023	Week 4	0.617096994	-0.209576198	0.820
1026	Week 4	0	-4	1.442
1028	Week 4	0	-4	1.442
1029	Week 4	0.001497813	-2.796473997	0.724

1031	Week 4	0.875412931	-0.057737435	0.911
1033	Week 4	0.010273005	-1.984095408	0.239
1036	Week 4	0.143374885	-0.843224113	0.442
1037	Week 4	3.350253807	0.525090672	1.259
1040	Week 4	0.01280041	-1.8893965	0.182
1042	Week 4	0.012833264	-1.888291863	0.182
1043	Week 4	0.008340052	-2.073654893	0.292
1046	Week 4	0.018092998	-1.740095728	0.093
1048	Week 4	9.93654651	0.99723984	1.540
1049	Week 4	0.037992317	-1.419162609	0.098
1052	Week 4	0.026154731	-1.580792421	0.002
1054	Week 4	57.81234808	1.76202136	1.997
1055	Week 4	0.400221399	-0.397591194	0.708
1058	Week 4	5.31996916	0.725917278	1.378
1059	Week 4	1.317161517	0.119672004	1.017
1062	Week 4	3.162639994	0.50006349	1.244
1064	Week 4	0	-4	1.442
1065	Week 4	0.00464231	-2.324010059	0.442
1068	Week 4	0.006734763	-2.165276566	0.347
1069	Week 4	0.013023886	-1.881937557	0.178
1071	Week 4	0.009797609	-2.00446969	0.251
1073	Week 4	1.352082207	0.131035217	1.023

Supplementary Table S7

Supplementary Table S7. Changes in *Bifidobacterium* and *Akkermansia* in the FAS population.

	**Run-In Mean (SD)**	**End of Study Mean (SD)**	***p* value** (Wilcoxon Signed-Rank test)	**Change Mean (SD)**	***p* value** (Brunner Munzel test)
Bifidobacterium					
Placebo	0.42 (0.96)	1.36 (5.9)	0.11	0.94 (5.89)	0.1
RPS	0.66 (2.67)	1.64 (5.5)	0.0004	0.98 (2.98)	
Akkermansia					
Placebo	0.90 (2.12)	2.91 (9.81)	0.12	2.01 (9.91)	0.37
RPS	1.36 (2.84)	1.74 (3.58)	0.14	0.38 (2.27)	

Within-group changes were compared using Wilcoxon Signed-Rank tests with Bonferroni correction and between-group changes were compared using Brunner-Munzel tests. Values shown are mean relative abundance with SD.

Supplementary Table S8. Z-scores derived from *Bifidobacterium* relative abundance values from the 2023 trial. Values with a Z-score greater than two are considered outliers and are indicated in bold.

Subject ID	Visit	Treatment	*Bifidobacterium*	Log10	Z-Score (Absolute value of ((x-Mean)/Standard Deviation))
1002	BL	3.5g	0.002335	-2.61352	0.078759
1005	BL	3.5g	0.000694	-3.10004	0.413223
1009	BL	3.5g	0.00062	-3.14241	0.456072
1010	BL	3.5g	0	-4	1.323286
1015	BL	3.5g	0	-4	1.323286
1017	BL	3.5g	0	-4	1.323286
1020	BL	3.5g	0.016052	-1.79177	0.909734
1023	BL	3.5g	0.000018	-3.92812	1.250597
1027	BL	3.5g	0.001387	-2.82757	0.137693
1028	BL	3.5g	0.07366	-1.13218	1.576723
1032	BL	3.5g	3.09E-05	-3.88306	1.205034
1036	BL	3.5g	0.00935	-2.02455	0.674338
1037	BL	3.5g	0.014299	-1.84168	0.859258
1040	BL	3.5g	0.000974	-2.96897	0.280687
1045	BL	3.5g	0.004493	-2.33791	0.357458
1047	BL	3.5g	0.002574	-2.5728	0.119931
1049	BL	3.5g	1.91E-05	-3.92409	1.246522
1052	BL	3.5g	0.036794	-1.43305	1.27248
1057	BL	3.5g	0.001603	-2.7688	0.078269
1062	BL	3.5g	5.52E-05	-3.80911	1.130251
1066	BL	3.5g	0.001572	-2.77682	0.086376
1067	BL	3.5g	0.000574	-3.17139	0.485379
1070	BL	3.5g	3.69E-05	-3.8636	1.185351
1075	BL	3.5g	0	-4	1.323286
1001	BL	Placebo	0	-4	1.323286
1006	BL	Placebo	0.000274	-3.42706	0.74391
1007	BL	Placebo	0.000436	-3.2712	0.586306
1011	BL	Placebo	0.001229	-2.87637	0.187046
1014	BL	Placebo	0.00018	-3.55271	0.870979
1018	BL	Placebo	0.000586	-3.16393	0.477831
1019	BL	Placebo	0.002768	-2.54239	0.150682
1024	BL	Placebo	0.006055	-2.21076	0.48604
1025	BL	Placebo	0.03232	-1.48919	1.215706
1030	BL	Placebo	0.00098	-2.96667	0.278356
1031	BL	Placebo	0.01694	-1.76853	0.933232
1034	BL	Placebo	0.011725	-1.9272	0.772777
1039	BL	Placebo	0.000761	-3.065	0.377786
1042	BL	Placebo	0.019861	-1.69983	1.002707
1044	BL	Placebo	9.84E-05	-3.70246	1.022404
1046	BL	Placebo	0	-4	1.323286
1050	BL	Placebo	6.71E-05	-3.77702	1.097807
1054	BL	Placebo	0.010268	-1.98429	0.715047

1055	BL	Placebo	0.067182	-1.1721	1.536352
1060	BL	Placebo	5.26E-05	-3.81645	1.137671
1064	BL	Placebo	5.02E-05	-3.82333	1.144633
1069	BL	Placebo	0.000791	-3.05014	0.362762
1072	BL	Placebo	0.000127	-3.64326	0.962537
1073	BL	Placebo	0.000297	-3.40159	0.718164
1002	Wk4	3.5g	0.008418	-2.06969	0.628693
1005	Wk4	3.5g	0.008929	-2.04438	0.654284
1009	Wk4	3.5g	0	-4	1.323286
1010	Wk4	3.5g	0.021978	-1.65604	1.046984
1015	Wk4	3.5g	0.021148	-1.67268	1.030156
1017	Wk4	3.5g	0.001155	-2.90139	0.212345
1020	Wk4	3.5g	0.001394	-2.82555	0.135652
1023	Wk4	3.5g	0.026354	-1.57751	1.126394
1027	Wk4	3.5g	0.001182	-2.8921	0.202951
1028	Wk4	3.5g	0	-4	1.323286
1032	Wk4	3.5g	0	-4	1.323286
1036	Wk4	3.5g	0.06605	-1.17947	1.528901
1037	Wk4	3.5g	0.092895	-1.03154	1.678495
1040	Wk4	3.5g	0.004739	-2.31526	0.380366
1045	Wk4	3.5g	0.072234	-1.14066	1.568154
1047	Wk4	3.5g	0.105865	-0.97484	1.735831
1049	Wk4	3.5g	0.091893	-1.03624	1.673738
1052	Wk4	3.5g	0.025405	-1.59338	1.110346
1057	Wk4	3.5g	0.038008	-1.41899	1.286696
1062	Wk4	3.5g	0.000651	-3.12431	0.437771
1066	Wk4	3.5g	2.63E-05	-3.8986	1.220744
1067	Wk4	3.5g	1.66E-05	-3.9333	1.255839
1070	Wk4	3.5g	0.016039	-1.79212	0.909379
1075	Wk4	3.5g	0.000136	-3.62686	0.945961
1001	Wk4	Placebo	0.009063	-2.03794	0.660796
1006	Wk4	Placebo	0.018182	-1.73798	0.964123
1007	Wk4	Placebo	0	-4	1.323286
1011	Wk4	Placebo	0	-4	1.323286
1014	Wk4	Placebo	4.65E-05	-3.83416	1.155587
1018	Wk4	Placebo	0.167465	-0.77582	1.937086
1019	Wk4	Placebo	0.056107	-1.25021	1.457369
1024	Wk4	Placebo	0.005833	-2.2267	0.469921
1025	Wk4	Placebo	0.009439	-2.02052	0.678418
1030	Wk4	Placebo	0.00013	-3.63776	0.956981
1031	Wk4	Placebo	0.00166	-2.75436	0.063668
1034	Wk4	Placebo	0.001513	-2.79226	0.101994
1039	Wk4	Placebo	2.98E-05	-3.88673	1.20874
1042	Wk4	Placebo	0.001569	-2.77757	0.087134
1044	Wk4	Placebo	0.042384	-1.37177	1.334443
1046	Wk4	Placebo	0.001064	-2.93416	0.24548
1050	Wk4	Placebo	0.015599	-1.80414	0.897221

1054	Wk4	Placebo	0.005431	-2.25718	0.439094
1055	Wk4	Placebo	0.00551	-2.25106	0.44528
1060	Wk4	Placebo	7.79E-05	-3.74982	1.070302
1064	Wk4	Placebo	0.03528	-1.45125	1.254077
1069	Wk4	Placebo	0.055479	-1.25509	1.452435
1072	Wk4	Placebo	0.002205	-2.63735	0.054658
1073	Wk4	Placebo	0.0103	-1.98298	0.716374

Supplementary Table S9

Supplementary Table S9. Z-scores derived from *Akkermansia* relative abundance values from the 2023 trial. Values with a Z-score greater than two are considered outliers and are indicated in bold.

Subject ID	Visit	Treatment	*Akkermansia*	Log10	Z-Score (Absolute value of ((x-Mean)/Standard Deviation))
1002	BL	3.5g	0.011167	-1.94819	0.471455
1005	BL	3.5g	3.86E-05	-3.85824	1.362129
1009	BL	3.5g	0.001241	-2.87262	0.415967
1010	BL	3.5g	0.07087	-1.14893	1.238726
1015	BL	3.5g	0.007742	-2.10555	0.320397
1017	BL	3.5g	0.005426	-2.25759	0.17444
1020	BL	3.5g	5.91E-05	-3.79833	1.304621
1023	BL	3.5g	0	-4	1.498218
1027	BL	3.5g	0.000353	-3.34375	0.868235
1028	BL	3.5g	0.00337	-2.45964	0.019514
1032	BL	3.5g	0.001297	-2.85471	0.398775
1036	BL	3.5g	9.26E-05	-3.71534	1.224956
1037	BL	3.5g	0.000039	-3.85699	1.360928
1040	BL	3.5g	0.002125	-2.65263	0.204782
1045	BL	3.5g	0.093036	-1.03088	1.352046
1047	BL	3.5g	0.002174	-2.64325	0.195778
1049	BL	3.5g	0.001832	-2.71394	0.263639
1052	BL	3.5g	4.21E-05	-3.84741	1.351732
1057	BL	3.5g	0.000153	-3.59746	1.111795
1062	BL	3.5g	0.017402	-1.7569	0.655088
1066	BL	3.5g	0.011887	-1.9213	0.497269
1067	BL	3.5g	0.038317	-1.41547	0.982852
1070	BL	3.5g	0.000554	-3.18451	0.715369
1075	BL	3.5g	0.004593	-2.32854	0.106331
1001	BL	Placebo	0.122659	-0.91095	1.467182
1006	BL	Placebo	0.15638	-0.80554	1.568368
1007	BL	Placebo	6.22E-05	-3.78995	1.296575
1011	BL	Placebo	0.011095	-1.95096	0.468797
1014	BL	Placebo	0.003151	-2.48792	0.046669
1018	BL	Placebo	0.000205	-3.51576	1.033359
1019	BL	Placebo	0.000142	-3.61626	1.129839
1024	BL	Placebo	0.000119	-3.66009	1.171916
1025	BL	Placebo	0.176772	-0.75234	1.619437

1030	BL	Placebo	0.018039	-1.74138	0.669991
1031	BL	Placebo	0.008835	-2.04891	0.374772
1034	BL	Placebo	5.05E-05	-3.82246	1.327788
1039	BL	Placebo	0.000211	-3.5067	1.02466
1042	BL	Placebo	0.035656	-1.44665	0.952923
1044	BL	Placebo	0.001477	-2.80224	0.348404
1046	BL	Placebo	0.045365	-1.34233	1.053069
1050	BL	Placebo	0.242033	-0.61595	1.750373
1054	BL	Placebo	0.003235	-2.47688	0.036064
1055	BL	Placebo	0.000201	-3.52101	1.038403
1060	BL	Placebo	0.008898	-2.04588	0.377683
1061	BL	Placebo			
1064	BL	Placebo	0.01871	-1.72561	0.685125
1069	BL	Placebo	0.000683	-3.10618	0.640175
1072	BL	Placebo	0.000127	-3.64326	1.155754
1073	BL	Placebo	0.000212	-3.506	1.02399
1002	Wk4	3.5g	0.119529	-0.92216	1.456412
1005	Wk4	3.5g	0.0625	-1.20343	1.18641
1009	Wk4	3.5g	0.049383	-1.30555	1.088377
1010	Wk4	3.5g	0.021978	-1.65604	0.751914
1015	Wk4	3.5g	0.07855	-1.1043	1.281566
1017	Wk4	3.5g	0.103364	-0.98521	1.395888
1020	Wk4	3.5g	0.000254	-3.45159	0.971759
1023	Wk4	3.5g	0.046826	-1.32859	1.066257
1027	Wk4	3.5g	0.037589	-1.42379	0.974867
1028	Wk4	3.5g	0.000221	-3.49393	1.012404
1032	Wk4	3.5g	0.000257	-3.44707	0.967424
1036	Wk4	3.5g	0	-4	1.498218
1037	Wk4	3.5g	0.022281	-1.65013	0.757591
1040	Wk4	3.5g	0.00206	-2.66547	0.217111
1045	Wk4	3.5g	0.002037	-2.6702	0.221649
1047	Wk4	3.5g	0.025487	-1.59199	0.813404
1049	Wk4	3.5g	0.000156	-3.59162	1.106186
1052	Wk4	3.5g	0.000102	-3.69371	1.204187
1057	Wk4	3.5g	0.119798	-0.92119	1.457349
1062	Wk4	3.5g	0.01971	-1.70312	0.706722
1066	Wk4	3.5g	0.000105	-3.68764	1.198358
1067	Wk4	3.5g	0.158273	-0.80032	1.573381
1070	Wk4	3.5g	0.00042	-3.28379	0.810682
1075	Wk4	3.5g	0.270157	-0.56822	1.796186
1001	Wk4	Placebo	0.045317	-1.34278	1.052635
1006	Wk4	Placebo	0.072727	-1.13771	1.249499
1007	Wk4	Placebo	0.051546	-1.28696	1.10622
1011	Wk4	Placebo	0.010526	-1.97362	0.447049
1014	Wk4	Placebo	0.000144	-3.61241	1.126139
1018	Wk4	Placebo	0.000254	-3.45138	0.971561
1019	Wk4	Placebo	0.000299	-3.39937	0.92163

1024	Wk4	Placebo	0.00053	-3.20045	0.730672
1025	Wk4	Placebo	0.017669	-1.75033	0.661401
1030	Wk4	Placebo	0.028573	-1.54253	0.860878
1031	Wk4	Placebo	0.060537	-1.21726	1.173128
1034	Wk4	Placebo	0.000146	-3.60826	1.12216
1039	Wk4	Placebo	0.003546	-2.43818	0.001079
1042	Wk4	Placebo	0.00109	-2.92463	0.465895
1044	Wk4	Placebo	0.043669	-1.35884	1.03722
1046	Wk4	Placebo	0.035811	-1.44477	0.954729
1050	Wk4	Placebo	0.055626	-1.25394	1.137916
1054	Wk4	Placebo	0.000397	-3.30329	0.829398
1055	Wk4	Placebo	0.001595	-2.77086	0.318277
1060	Wk4	Placebo	0.002531	-2.57989	0.134951
1064	Wk4	Placebo	0.001313	-2.84981	0.394074
1069	Wk4	Placebo	6.12E-05	-3.79263	1.299154
1072	Wk4	Placebo	0.00017	-3.56927	1.084729
1073	Wk4	Placebo	0.000767	-3.06198	0.597749
